# Quantitative Biomarkers, Genomic Assays, and Demographics Associated with Breast-Conserving Surgery Following Neoadjuvant Therapy in Early-Stage, Hormone Receptor-Positive, HER-Negative Breast Cancer

**DOI:** 10.1245/s10434-024-16160-5

**Published:** 2024-09-09

**Authors:** Jincong Q. Freeman, Sarah P. Shubeck, Nan Chen, Sudha R. Yarlagadda, Rita Nanda, Dezheng Huo, Frederick M. Howard

**Affiliations:** 1 Department of Public Health Sciences, University of Chicago, Chicago, IL; 2 Cancer Prevention and Control Program, UChicago Medicine Comprehensive Cancer Center, Chicago, IL; 3 Center for Health and the Social Sciences, University of Chicago, Chicago, IL; 4 Department of Surgery, University of Chicago Medicine, Chicago, IL; 5 Section of Hematology/Oncology, Department of Medicine, University of Chicago, Chicago, IL; 6 Center for Clinical Cancer Genetics and Global Health, University of Chicago, Chicago, IL

**Keywords:** Neoadjuvant therapy, Quantitative biomarkers, Genomic assays, Breast-conserving surgery, Racial disparities, Early-stage breast cancer, National Cancer Database

## Abstract

**Background.:**

Given increased neoadjuvant therapy use in early-stage, hormone receptor (HR)-positive/HER2-negative breast cancer, we sought to quantify likelihood of breast-conserving surgery (BCS) after neoadjuvant chemotherapy (NACT) or endocrine therapy (NET) as a function of ER%/PR%/Ki-67%, 21-gene recurrence scores (RS), or 70-gene risk groups.

**Methods.:**

We analyzed the 2010–2020 National Cancer Database. Surgery was categorized as “mastectomy/BCS.” Logistic regression was performed. Adjusted odds ratios (AOR) were per 10-unit increase in ER%/PR%/Ki-67%.

**Results.:**

Overall, 42.3% underwent BCS after NACT, whereas 64.0% did after NET. Increasing ER% (AOR = 0.96, 95% confidence interval [CI] 0.94–0.97) or PR% (AOR=0.98, 95% CI 0.96–0.99) was associated with lower odds of BCS after NACT. Increasing Ki-67% was associated with greater odds of BCS (AOR = 1.07, 95% CI 1.04–1.10). Breast-conserving surgery rates increased by ~20 percentage points, with Ki-67% ≥15 or RS >20. Patients with a low (43.0%, AOR = 0.50, 95% CI 0.29–0.88) or intermediate (46.4%, AOR = 0.58, 95% CI 0.41–0.81) RS were less likely than patients with a high RS (65.0%) to undergo BCS after NACT. Increasing ER% was associated with higher odds of BCS after NET (AOR = 1.09, 95% CI 1.01–1.17). Breast-conserving surgery rates increased by ~20 percentage points between ER <50% and >80%. In both cohorts, the odds of BCS were similar between 70-gene low-risk and high-risk groups. Asian or uninsured patients had lower odds of BCS.

**Conclusions.:**

Neoadjuvant chemotherapy is unlikely to downstage tumors with a low-intermediate RS, higher ER%/PR%, or lower Ki-67%. Breast-conserving surgery after NET was most dependent on ER%. Findings could facilitate treatment decision-making based on tumor biology and racial/socioeconomic disparities and improve patient counseling on the likelihood of successful BCS.

Breast cancer treatment options are driven by tumor characteristics, including stage and molecular subtype. In the United States, 70% of breast cancers are hormone receptor (HR)-positive and human epidermal growth factor receptor 2 (HER2)-negative, and up to one fourth of patients will present with locally advanced disease.^1,2^ Neoadjuvant chemotherapy (NACT) and neoadjuvant endocrine therapy (NET) often are used to downstage locally advanced tumors and/or allow for breast-conserving surgery (BCS) among patients with early-stage, HR-positive/HER2-negative breast cancer that may otherwise require mastectomy. In recent years, both use of NACT and NET have increased in clinical practice.^3,4^ Pathological complete response (pCR), referring to the absence of invasive tumor in the breast and/or axillary lymph nodes based on the histopathologic examination (i.e., ypT0/Tis ypN0),^5^ is a common endpoint in studies of triple-negative and HER2-positive breast cancer. However, pCR is uncommon after neoadjuvant systemic therapy for HR-positive/HER2-negative disease—occurring in only about 10% or less of this patient population.^6^ Thus, downstaging to allow for BCS or deem matted axillary nodes operable may be more attainable endpoints after neoadjuvant treatment of HR-positive/HER2-negative breast cancers.

According to the American Society of Clinical Oncology practice guidelines, molecular markers including estrogen receptor (ER), progesterone receptor (PR), and HER2 expression should be routinely used to guide treatment decisions in the neoadjuvant setting, and these markers are all associated with response to NACT or NET.^7,8^ Ki-67, a biomarker for cellular proliferation, predicts response to NACT^9^ and has been confirmed to predict recurrence-free survival after NET^10^ and aid adjuvant therapy selection among postmenopausal women with stage I-III, HR-positive breast cancer in randomized trials.^11^ Meta-analyses also have reported that a higher level of Ki-67 expression is associated with worse overall survival among patients with early-stage breast cancer, regardless of nodal status.^12,13^ Moreover, genomic assays, e.g., the 21-gene and the 70-gene assays, are helpful tools in guiding neoadjuvant treatment decision-making, as their risk scores have been found to predict pCR after NACT among patients with early-stage, HR-positive/HER2-negative breast cancer.^14–16^ Additionally, previous research has demonstrated the predictive value of the 21-gene recurrence score (RS) on pCR rate for patients with early-stage, HR-positive/HER2-negative breast cancer who receive NET.^17^

While the rate of pCR is usually low among patients with early-stage, HR-positive/HER2-negative breast cancer who receive NACT or NET, achieving pCR does not translate directly into BCS. Furthermore, data are limited on quantitative biomarkers and genomic assays predicting BCS in this patient population. Estrogen receptor and PR status are mainly assessed as binary categories: negative and positive. A recent study has found that increasing ER percentage is associated with a lower likelihood of tumor downstage and pCR at surgery among patients who receive NACT.^18^ In the era of precision medicine, incorporating quantitative assessments of ER, PR, and Ki-67 expression, the 21-gene RS, or the 70-gene risk scores could help clinicians identify and further classify patient groups with regard to tumor response and neoadjuvant treatment selection as well as subsequent BCS. Given the increasing use of neoadjuvant systemic therapy and genomic assays for early-stage, HR-positive/HER2-negative breast cancer,^3,4,19^ we sought to quantify the success of NACT or NET for surgical plan (BCS vs. mastectomy) as a function of ER, PR, and Ki-67 expression, the 21-gene RS, or 70-gene risk scores in this patient population, using a large, real-world U.S. oncology database.

## METHODS

### Study Design and Data Source

This was a retrospective study. We analyzed data obtained from patients with stage I-III, HR-positive/HER2-negative breast cancer diagnosed from 2010 to 2020 in the National Cancer Database (NCDB). The NCDB is a joint project of the Commission of Cancer of the American College of Surgeons and the American Cancer Society; it is a clinical oncology registry that collects data from more than 1500 Commission of Cancer-accredited cancer programs, capturing approximately 72.0% of new cancer diagnoses in the United States each year.^20–22^ Complying with the NCDB’s Data Use Agreement, we suppressed reporting of cell counts less than 10 to protect patients’ confidentiality. Informed consent was not required for the use of deidentified patient-level data, and the University of Chicago Institutional Review Board determined that the current study was exempt from review. This study followed the Strengthening the Reporting of Observational Studies in Epidemiology (STROBE) reporting guideline.^23^

### Eligibility and Cohort Selection

Supplementary Fig. 1 illustrates the selection of patient cohorts. Briefly, patients were eligible if they were at least 18 years of age at diagnosis and had stage I, II, or III disease, with a molecular subtype of HR-positive/HER2-negative. This study included patients who received either NACT or NET. For the NACT cohort, we restricted it to patients who received the treatment for 12–30 weeks before their surgeries as they were likely to complete the treatment and achieve pCR (Supplementary Fig. 2A). The NET cohort consisted of patients who completed the treatment for 4–36 months before surgery owing to a greater likelihood of treatment completion and pCR (Supplementary Fig. 2B).

### Variables and Measures

The primary outcome was surgical plan, which was dichotomized as mastectomy and BCS. The independent variables included quantitative biomarkers and genomic assay testing results. Quantitative data on ER, PR, and Ki-67 expression became available after 2017, which were analyzed in subgroup analyses. Percent positive, ranging from 0 to 100, was recorded for these three biomarkers in the NCDB. We also categorized PR expression into three groups: 0%, 1–10%, and >10%. Ki-67 expression categories included <10%, 10–20%, and >20%. The 21-gene RS was assessed as a continuous variable (between 0 and 100) and further categorized as low risk (0–10), intermediate risk (11–25), or high risk (≥26) as per the TAILORx trial.^24^ The 70-gene risk scores were dichotomized into low-risk and high-risk groups, and the numeric values were not available in the NCDB.

Covariates assessed were age at diagnosis, age group (≤50 years and >50 years), year of initial cancer diagnosis, race/ethnicity (non-Hispanic [NH] Asian, NH Black, Hispanic, NH White, and other/multiracial), primary payer at diagnosis (uninsured, private, Medicaid, Medicare, and other/unknown), type of facility, Charlson-Deyo Comorbidity Index, histologic type (ductal, lobular, both ductal and lobular, and other), HER2 IHC score (0, 1+, and 2+), AJCC clinical T stage (cT1-cT4), AJCC clinical nodal status (negative [cN0] and positive [cN1+]), and tumor grade (1–3).

### Statistical Analysis

To describe patient cohorts, we calculated descriptive statistics and compared the distributions of the characteristics by neoadjuvant therapy using bivariate analysis. *P* values were computed by using Student’s *t*, Wilcoxon rank-sum, Pearson’s chi-squared, or Fisher’s exact tests, as appropriate. To assess demographic and clinicopathologic characteristics associated with surgical plan by treatment cohort, we conducted bivariate analyses followed by multivariable logistic regression. Predicted rates of BCS by ER, PR, or Ki-67 expression, and continuous 21-gene RS were estimated by using restricted cubic spline logistic regression. Then, multivariable logistic regression models were fit separately to examine the associations between quantitative biomarkers and surgical plan or between genomic assay results and surgical plan for the two treatment cohorts. Adjusted odds ratios (AOR) and 95% confidence intervals (CI) were calculated. The AORs and corresponding 95% CIs for percentages of ER, PR, and Ki-67 expression were per 10-unit increase. A two-sided *P* value <0.05 was considered statistically significant, and all data analyses were performed using Stata 17 (StataCorp, College Station, TX).

## RESULTS

### NACT Cohort

Of 44,589 patients treated with NACT, the mean age was 53.1 years (SD 11.8), the median amount of time they were on NACT before surgery was 21.4 weeks (interquartile range 18.6–24.7), and 65.1% had cT1-2 disease (Supplementary Table 1). Overall, 42.3% of the patients underwent BCS after NACT (Table 1), whereas the proportion (57.0%) was higher among those who achieved pCR (Supplementary Table 2). Compared with NH White patients, NH Black patients had higher odds of BCS (AOR 1.12, 95% confidence interval [CI] 1.03–1.21), whereas NH Asian patients had lower odds of BCS (AOR 0.80, 95% CI 0.70–0.91). Uninsured patients (AOR 0.84, 95% CI0 0.71–0.98) or patients with Medicare (AOR 0.90, 95% CI 0.83–0.99) were less likely than those privately insured to have undergone BCS after NACT (Table 1).

**FIG. 1:**
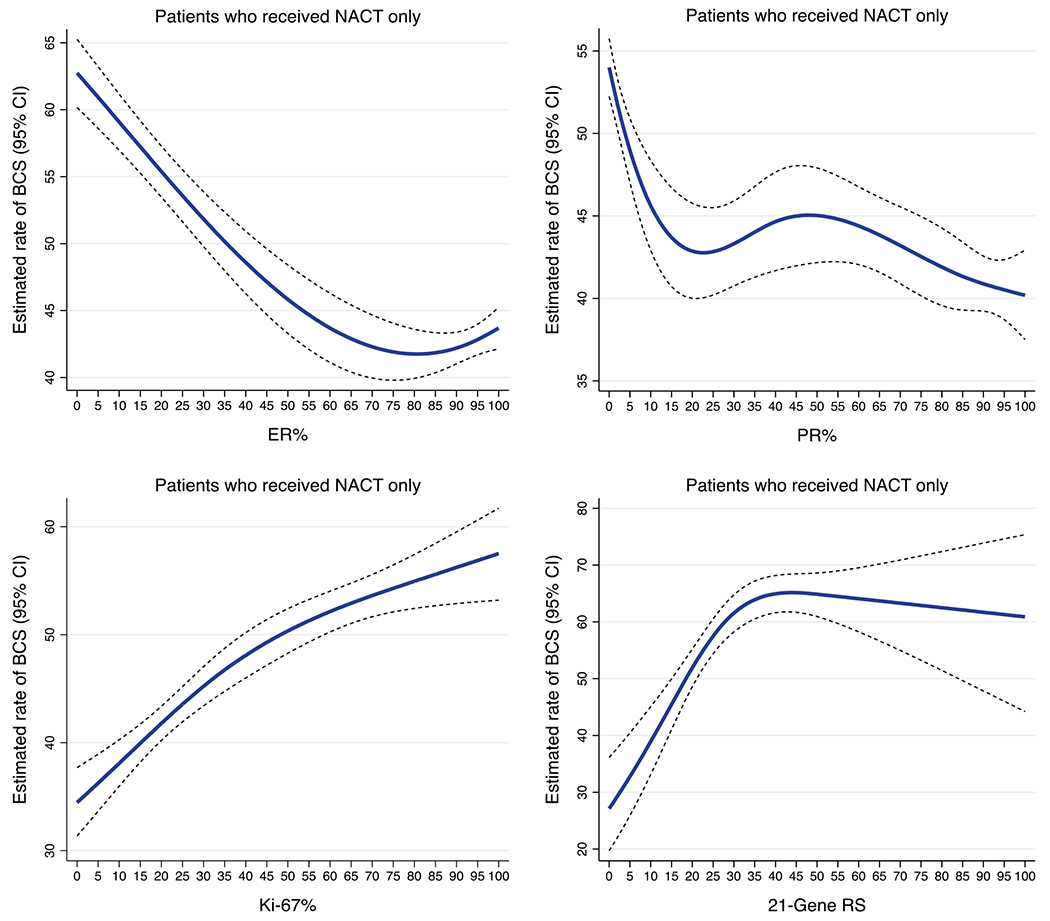
Estimated rates of BCS in early-stage, HR-positive/HER2-negative breast cancer patients who received NACT, by quantitative biomarkers and 21-gene recurrence scores. Blue lines represent estimated rates of BCS. Dash lines represent the corresponding 95% CIs. *BCS* breast-conserving surgery; *HR* hormone receptor; *HER2* human epidermal growth factor receptor 2; *NACT* neoadjuvant chemotherapy; *CI* confidence interval; *HR* estrogen receptor; *PR* progesterone receptor; *RS* recurrence score

For the subgroup analysis, 13,752 patients with available quantitative biomarker data were included. Figure 1 shows the estimated BCS rates by ER%, PR%, Ki-67%, and continuous 21-gene RS. Particularly, the BCS rate increased by approximately 20 percentage points with Ki-67 expression being ≥15% or 21-gene RS of >20. Similar patterns were observed among patients with locally advanced tumors (cT3-cT4), although there were lower rates of BCS overall (between 20.0–40.0%) in this subgroup (Supplementary Fig. 3A). After controlling for demographic and clinicopathologic factors (Table 2), increasing ER expression (AOR 0.96, 95% CI 0.94–0.97) and PR expression (AOR 0.98, 95% CI 0.96–0.99) were associated with lower odds of BCS. Increasing Ki-67 expression was associated with greater odds of BCS (AOR 1.07, 95% CI 1.04–1.10). We observed similar patterns in the models with categorical PR or Ki-67 expression (Table 2). Patients with a low RS (AOR 0.50, 95% CI 0.29–0.88) or intermediate RS (AOR 0.58, 95% CI 0.41–0.81) were significantly less likely than patients with a high RS to have undergone BCS after NACT. Patients in the 70-gene high-risk group were numerically more likely than those in the low-risk group to have had BCS, although not statistically significant (AOR 1.17, 95% CI 0.66–2.08) (Table 2).

### NET Cohort

Of 10,466 patients treated with NET, the mean age was 68.4 years (SD 11.5), the median amount of time they were on NET before surgery was 6.3 months (interquartile range 5.1–8.2), and 76.4% had cT1-cT2 disease (Supplementary Table 1). Overall, 64.0% of the patients underwent BCS after NET (Table 3), with a slightly higher proportion (67.3%) among those who achieved pCR (Supplementary Table 2). NH Asian patients were less likely than NH White patients to have had BCS after NET (AOR 0.69, 95% CI 0.53–0.91). Patients without insurance had lower odds of BCS than those with private insurance (AOR 0.59, 95% CI 0.43–0.82) (Table 3).

**FIG. 2:**
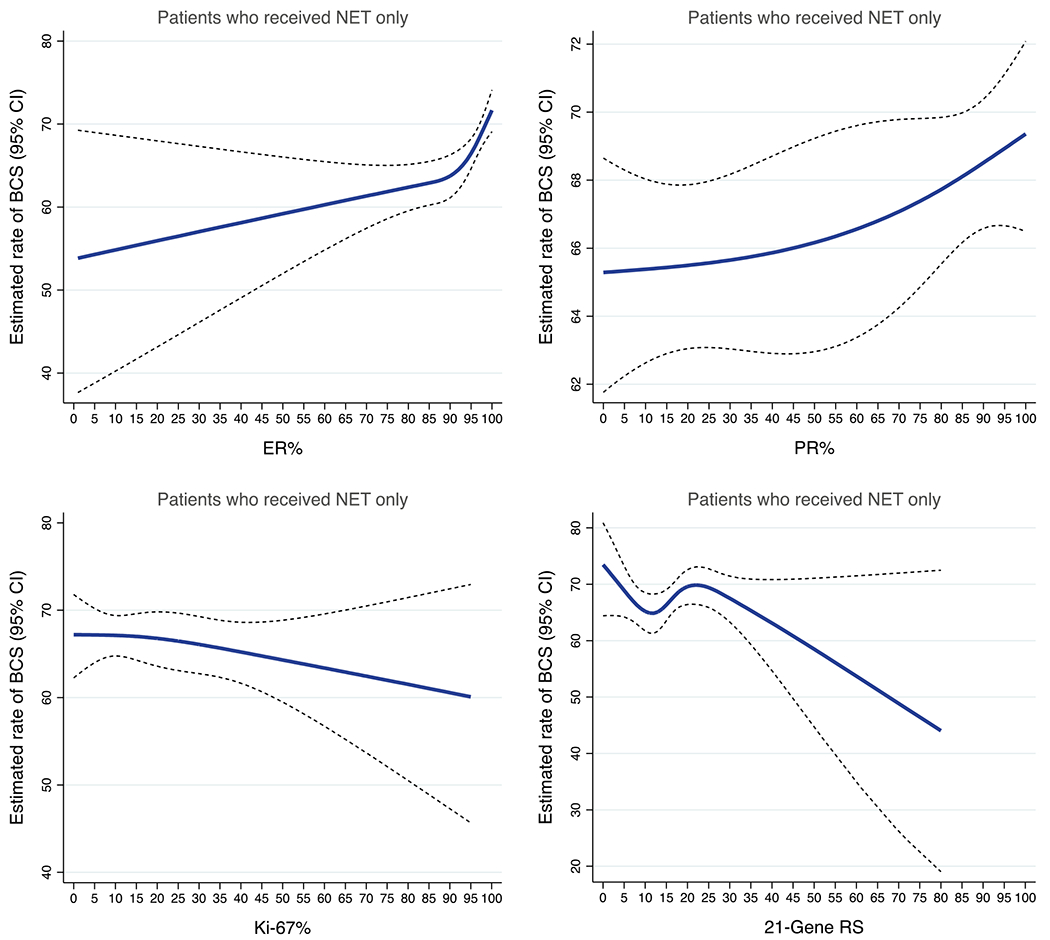
Estimated rates of BCS in early-stage, HR-positive/HER2-negative breast cancer patients who received NET, by quantitative biomarkers and 21-gene recurrence scores. Blue lines represent estimated rates of BCS. Dash lines represent the corresponding 95% CIs. *BCS* breast-conserving surgery; *HR* hormone receptor; *HER2* human epidermal growth factor receptor 2; *NET* neoadjuvant endocrine therapy; *CI* confidence interval; *HR* estrogen receptor; *PR* progesterone receptor; *RS* recurrence score

For the subgroup analysis, 4003 patients with available quantitative biomarker data were included. The estimated rates of BCS by ER%, PR%, Ki-67%, and continuous 21-gene RS are depicted in Fig. 2. In particular, the rate of BCS increased by approximately 20 percentage points, when ER expression was >80%. We observed similar patterns in the subgroup of patients with locally advanced tumors (cT3-cT4) but with a 20.0–40.0% rate of BCS (Supplementary Fig. 3B). After covariate adjustment (Table 4), increasing ER% was associated with greater odds of BCS (AOR 1.09, 95% CI 1.01–1.17). There was no significant association between baseline PR expression and BCS (AOR 1.02, 95% CI 0.99–1.04) or between Ki-67 expression and BCS (AOR 0.98, 95% CI 0.91–1.05) after NET. However, compared with the 0% PR expression category, the odds of BCS were higher for the 1–10% category (AOR 1.46, 95% CI 1.01–2.10) or the >10% category (AOR 1.30, 95% CI 1.02–1.66). The odds of BCS after NET were greater for patients in the >20% Ki-67 expression category than the 10–20% category (AOR 1.21, 95% CI 1.01–1.45) (Table 4). Patients with a low (AOR 0.92, 95% CI 0.60–1.43) or intermediate (AOR 0.96, 95% CI 0.65–1.41) RS were numerically less likely than patients with a high RS to have undergone BCS, although not statistically significant. The odds of BCS were not significantly different between the 70-gene high-risk group and the low-risk group (AOR 0.94, 95% CI 0.43–2.04) (Table 4).

Furthermore, we observed that patient characteristics differed significantly by treatment cohort and by pCR status (Supplementary Table 3). Specifically, compared with patients who did not achieve pCR, those who did had a lower mean ER expression (42.9% vs. 82.3%, *P* < 0.001) and PR expression (19.4% vs. 51.0%, *P* < 0.001), a higher mean Ki-67 expression (59.1% vs. 32.5%, *P* < 0.001), with a larger proportion having cN0 (57.6% vs. 49.0%, *P* < 0.001). Patients who achieved pCR were more likely to have a high 21-gene RS than those who did not (78.9% vs. 33.3%, *P* < 0.001). In addition, the proportion of BCS was higher among patients who achieved pCR than among those who did not (57.5% vs. 46.3%, *P* < 0.001) (Supplementary Table 3).

## DISCUSSION

In this study of patients with early-stage, HR-positive/HER2-negative breast cancer from the NCDB, higher ER or PR expression and lower Ki-67 expression were associated with a lower likelihood of BCS after NACT. Among patients who received NET, higher ER expression was associated with a higher likelihood of BCS. In both treatment cohorts, the rate of BCS was lower among NH Asian patients than their NH White counterparts, and patients without insurance also had a lower BCS rate than those privately insured.

Consistent with previous studies,^25–27^ more than one-third of the patients underwent BCS after NACT. Importantly, we showed that the rate of BCS was higher among patients with higher Ki-67% but was lower among those with higher ER or PR expression. Previous research has established the predictability of pCR or downstaging tumors to enable BCS after NACT for patients with negative ER or PR status.^28,29^ Raphael et al. reported lower odds of tumor response and pCR at surgery with increasing ER percent positive among patients who receive NACT.^18^ The current study further depicts inverse associations between continuous ER and PR expression and BCS and a positive association between Ki-67 expression and BCS. NACT may be more effective for patients with <20% ER positivity, <15% PR positivity, or ≥15% Ki-67 positivity. Our findings provide evidence that NACT is likely to downstage tumors, allowing for BCS with lower ER and PR expression or higher Ki-67 expression and increasing the likelihood of BCS in early-stage, HR-positive/HER2-negative breast cancer that may otherwise require a mastectomy.

In the NET cohort, 64.0% of patients underwent BCS, which is congruent with the estimated rates documented in the literature.^30–32^ This high BCS rate is likely owing to smaller clinical tumor size, as 76.4% of patients in the NET cohort were diagnosed with cT1-cT2 disease. It also is because these patients were mostly older or frail, given an average age of 68.4 years at diagnosis observed in this NET cohort. Because we included patients treated with NET for up to 36 months, these patients may eventually have undergone BCS. In this cohort, we observed that only 2.7% of the patients received more than 1 year of NET and pCR rates were similar in 1–3 years, with age at diagnosis being fairly uniform. We found a significantly greater likelihood of BCS as ER percent positive increased. For quantitative PR and Ki-67 assessments, their relationships with BCS may not be linear, because the likelihood of BCS after NET is not statistically significant. Interestingly, when comparing categorical PR expression, patients in the 1–10% or >10% group were more likely to undergo BCS, which suggests the homogeneity of BCS rate in these two groups. Having >20% of Ki-67 expression was associated with greater odds of BCS. These findings suggest that BCS after NET was most dependent on ER expression in early-stage, HR-positive/HER2-negative breast cancer, and NET may be more effective when ER positivity is greater than 50. The nonlinear relationship between PR expression and BCS or between Ki-67 expression and BCS after NET in this patient population is worth further investigation and validation.

Genomic assay testing results, in addition to the assessments of quantitative ER, PR, and Ki-67 expression, can be beneficial to neoadjuvant treatment decision-making. We found that patients with a low or intermediate 21-gene RS were less likely than those with a high RS to have undergone BCS after NACT. In the NET cohort, we observed numerically lower rates of BCS in the low or intermediate 21-gene RS groups, although not statistically significant. The results suggest that neoadjuvant systemic therapy is unlikely to shrink tumors with a low or intermediate 21-gene RS. In clinical practice, neoadjuvant systemic therapy often is considered for node-positive patients with HR-positive/HER2-negative disease even without the results of genomic assay testing, but our findings highlight that genomic testing results may be used to avoid the unnecessary or ineffective use of chemotherapy in the neoadjuvant setting. It is probably explained by lower rates of pCR among patients in these two RS categories based on the existing literature.^14–16^ Our data suggest that the 21-gene assay may help to inform the likelihood of successfully downstaging tumors to allow for BCS after neoadjuvant systemic therapy. However, the BCS rates were found to be similar comparing the 70-gene low-risk group to the high-risk group, probably owing to the small sample size. Therefore, future research is needed to determine the association between the 70-gene assay and BCS after neoadjuvant systemic therapy.

Other important findings pertain to racial and socioeconomic disparities in BCS among patients with early-stage, HR-positive/HER2-negative breast cancer who received neoadjuvant systemic therapy. Compared with NH White patients, NH Asian patients were 20–31% less likely to have undergone BCS after NACT or NET; in contrast, NH Black patients were 12% more likely to have undergone BCS after NACT. This finding is in line with previous studies.^33–35^ Thomas et al. reported that Asian or Pacific Islander breast cancer patients had 26% lower odds of BCS, whereas NH Black patients had a 23% greater likelihood of undergoing BCS than their NH White counterparts.^33^ Similarly, a recent analysis has revealed that Asian American, Native Hawaiian, and Pacific Islander women with cT1-2N0M0 breast cancer are 1.35 times more likely than White women to undergo mastectomy (vs. BCS), and Black patients are less likely to do so.^35^ These racial disparities in BCS rates may be due to differential tumor sizes, patient preferences, fear, and cultural beliefs.^36^ Regarding primary payer at diagnosis, we found that uninsured patients and Medicare enrollees were 16% and 10% less likely than those privately insured to have had BCS after NACT, respectively. In the NET cohort, patients without insurance had lower odds of BCS than those with private insurance. Our results in the neoadjuvant setting are consistent with the existing literature.^33,35,37,38^ For example, uninsured patients and those on Medicaid or Medicare are 16–25% less likely than those with private insurance to undergo BCS for cT1-T2N0-3 breast cancer.^37^ In conclusion, these data suggest the potential need for oncology programs to identify unmet needs and ensure equitable access to BCS while adequately addressing the disparities in this patient population.

Several limitations of this study should be noted. Most importantly, there are a number of reasons for considering mastectomy, irrespective of treatment response, including germline mutational status or patient preference to avoid adjuvant radiation therapy. Although we presume the differing rates of BCS as a function of biomarkers is due to response to neoadjuvant systemic therapies, the nuances of these treatment decisions are not recorded in the NCDB. Second, because of the retrospective nature of the NCDB registry, we were not able to accurately evaluate how clinicians selected the patients for their neoadjuvant treatments, and treatment misclassification is possible. Third, the NCDB does not collect information on specific types of NACT or NET regimens, which may influence the estimated rates of BCS. The registry also does not collect data on the timing of Ki-67 and other biomarkers, which can change after neoadjuvant treatment with such changes predicting treatment response and risk of recurrence. It is worth exploring in future research whether such dynamics have an impact on the likelihood of BCS after neoadjuvant systemic therapy. Additionally, there are unmeasured sociodemographic and clinicopathologic factors that probably affect the associations observed in the current study. The two treatment cohorts might not represent all patients with early-stage, HR-positive/HER2-negative breast cancer. Prospective or randomized trials are needed to validate the utility of quantitative ER, PR, and Ki-67 expression, the 21-gene RS, and the 70-gene risk scores predicting BCS after NACT or NET in clinical practice.

## CONCLUSIONS

In this real-world data analysis of NCDB patients who received neoadjuvant systemic therapy for early-stage, HR-positive/HER2-negative breast cancer, the rate of BCS after NACT was higher among patients with a high 21-gene RS, lower ER/PR expression, or higher Ki-67 expression. Most patients who received NET underwent BCS; BCS after NET was most dependent on ER expression. NH Asian patients were less likely than their NH White counterparts to have undergone BCS after NACT or NET. Lack of health insurance coverage was associated with a lower likelihood of BCS in both treatment cohorts. These data could facilitate neoadjuvant treatment decision-making and optimal patient selection for surgery based on tumor biology and improve patient counseling on the likelihood of successful BCS. Meanwhile, it is also critical for breast oncology programs to ensure equitable care access and address racial and socioeconomic BCS disparities in this patient population.

## Supplementary Material

Supplemental Materias

## Figures and Tables

**TABLE 1:** Associated characteristics with surgical plan (breast-conserving surgery vs. mastectomy) in early-stage, HR-positive/HER2-negative breast cancer patients who received neoadjuvant chemotherapy

Overall	Among patients who received NACT only^a^			
	Surgical plan		*P* ^b^	Multivariable logistic regression
	Mastectomy	Breast-conserving surgery		
	*n* = 19,011 (57.7%)	*n* = 13,933 (42.3%)		
	No. (%)	No. (%)		AOR (95% CI)
Age at diagnosis (years)				
Mean (SD)	54.3 (12.0)	54.8 (11.1)	<0.001	**1.07 (1.03–1.11)** ^§,d^
Median (IQR)	54.0 (46.0, 63.0)	55.0 (47.0, 63.0)	<0.001	
Age group (years)				
≤50	7391 (38.9)	5023 (36.1)	<0.001	–
>50	11,620 (61.1)	8910 (63.9)		–
Race/ethnicity^c^				
Non-Hispanic white	12,755 (67.1)	9354 (67.1)	0.004	1.0 (reference)
Non-Hispanic black	2805 (14.8)	2226 (16.0)		**1.12 (1.03–1.21)** ^†^
Non-Hispanic Asian	963 (5.1)	659 (4.7)		**0.80 (0.70–0.91)** ^†^
Hispanic	2000 (10.5)	1362 (9.8)		0.95 (0.86–1.05)
Other	488 (2.6)	332 (2.4)		0.90 (0.74–1.09)
Primary payer at diagnosis				
Uninsured	830 (4.4)	479 (3.4)	<0.001	**0.84 (0.71–0.98)**
Private/managed care	11,182 (58.8)	8608 (61.8)		1.0 (reference)
Medicaid	2370 (12.5)	1578 (11.3)		0.95 (0.86–1.04)
Medicare	4114 (21.6)	2884 (20.7)		**0.90 (0.83–0.99)**
Other government/unknown	515 (2.7)	384 (2.8)		1.01 (0.84–1.21)
Facility type/cancer program				
Community	1119 (6.7)	827 (6.5)	0.011	**1.15 (1.01–1.30)**
Comprehensive community	6314 (37.7)	4964 (39.1)		**1.13 (1.06–1.21)** ^§^
Academic/research	6004 (35.9)	4215 (33.2)		1.0 (reference)
Integrated network	3304 (19.7)	2688 (21.2)		**1.16 (1.07–1.26)** ^§^
Charlson-Deyo Comorbidity Index				
0	16332 (85.9)	12,120 (87.0)	0.019	1.0 (reference)
1	2126 (11.2)	1436 (10.3)		0.91 (0.83–1.00)
≥2	553 (2.9)	377 (2.7)		0.88 (0.74–1.04)
Histologic type				
Ductal	13,635 (71.7)	12,172 (87.4)	<0.001	1.0 (reference)
Lobular	2430 (12.8)	856 (6.1)		**0.48 (0.43–0.53)** ^§^
Ductal and lobular	997 (5.2)	527 (3.8)		**0.72 (0.63–0.83)** ^§^
Other	1949 (10.3)	378 (2.7)		**0.47 (0.40–0.56)** ^§^
AJCC stage group				
I	2448 (12.9)	2321 (16.7)	<0.001	–
II	8582 (45.1)	9313 (66.8)		–
III	7981 (42.0)	2299 (16.5)		–
AJCC clinical T stage				
cT1	2941 (15.6)	3074 (22.1)	<0.001	1.0 (reference)
cT2	7134 (37.7)	8592 (61.9)		**1.10 (1.02–1.18)**
cT3	4984 (26.4)	1748 (12.6)		**0.35 (0.32–0.38)** ^§^
cT4	3848 (20.4)	470 (3.4)		**0.13 (0.11–0.15)** ^§^
AJCC clinical nodal status				
Negative (cN0)	7383 (39.1)	6889 (49.7)	<0.001	**1.29 (1.21–1.36)** ^§^
Positive (cN1+)	11,496 (60.9)	6975 (50.3)		1.0 (reference)
PR status				
Negative	3875 (20.4)	3773 (27.1)	<0.001	**1.22 (1.13–1.30)** ^§^
Positive	15,113 (79.6)	10,145 (72.9)		1.0 (reference)
HER2 IHC				
Score 0	4373 (28.6)	3432 (30.2)	<0.001	1.0 (reference)
Score 1+	6889 (45.1)	4820 (42.4)		**0.88 (0.82–0.94)** ^§^
Score 2+	4006 (26.2)	3104 (27.3)		0.98 (0.90–1.06)
Tumor grade				
1	1999 (11.2)	1137 (8.6)	<0.001	**0.62 (0.55–0.68)** ^§^
2	8902 (50.1)	5757 (43.4)		**0.76 (0.71–0.81)** ^§^
3	6879 (38.7)	6371 (48.0)		1.0 (reference)
Days of NACT before surgery				
Mean (SD)	150.3 (29.7)	148.2 (30.5)	<0.001	–
Median (IQR)	152.0 (130.0, 173.0)	149.0 (127.0, 172.0)	<0.001	–

Bold values indicate significant findings at *P* < 0.05

*HR* hormone receptor; *HER2* human epidermal growth factor receptor 2; *NACT* neoadjuvant chemotherapy; *SD* standard deviation; *IQR* interquartile range; *PR* progesterone receptor; IHC, immunohistochemistry; *AJCC* American Joint Committee on Cancer; *AOR* adjusted odds ratio; *CI* confidence interval

a NACT cohort included patients who started NACT for 12–30 weeks before surgery

b *P* values were computed by using Student’s *t*, Wilcoxon rank-sum, or Pearson’s chi-squared tests as appropriate

c Other includes American Indian, Alaska Native, Hawaiian and other Pacific Islander, other, or unknown races/ethnicities

d AOR per 10-year increase for age at diagnosis

† *P* < 0.005;

§ *P* < 0.001

**TABLE 2:** Subgroup analyses of surgical plan in early-stage, HR-positive/HER2-negative breast cancer patients who received neoadjuvant chemotherapy, with available quantitative biomarker and genomic assay data

	Among patients who received NACT only^a^			
	Surgical plan		*P* ^b^	Multivariable logistic regression
	Mastectomy	Breast-conserving surgery	
	No. (row %)	No. (row %)		AOR (95% CI)
ER%, mean (SD)	78.7 (31.0)	69.9 (37.4)	<0.001	**0.96 (0.94–0.97)** ^c,d,§^
PR%, mean (SD)	47.7 (39.9)	39.8 (39.9)	<0.001	0.98 (0.96–0.99)^c,d,§^
PR% category				
0%	1228 (45.3)	1483 (54.7)	<0.001	1.0 (reference)
1–10%	643 (53.2)	565 (46.8)		**0.75 (0.64–0.89)** ^c,†^
>10%	17,140 (59.0)	11,885 (41.0)		**0.74 (0.67–0.82)** ^c,§^
Ki-67%, mean (SD)	35.9 (26.5)	43.0 (27.6)	<0.001	**1.07 (1.04–1.10)** ^c,d,§^
Ki-67% category				
<10%	460 (65.4)	243 (34.6)	<0.001	1.0 (reference)
10–20%	716 (59.7)	484 (40.3)		**1.39 (1.10–1.75)** ^c,†^
>20%	17,835 (57.5)	13,206 (42.5)		**1.50 (1.24–1.80)** ^c,§^
21-gene recurrence score				
Low (0–10)	49 (57.0)	37 (43.0)	<0.001	**0.50 (0.29–0.88)** ^e^
Intermediate (11–25)	202 (53.6)	175 (46.4)		**0.58 (0.41–0.81)** ^e,†^
High (26–100)	307 (35.0)	571 (65.0)		1.0 (reference)
70-gene risk group				
Low risk	56 (51.4)	53 (48.6)	0.021	1.0 (reference)
High risk	231 (39.5)	354 (60.5)		1.17 (0.66–2.08)^e^

Bold values indicate significant findings at *P* < 0.05

*HR* hormone receptor; *HER2* human epidermal growth factor receptor 2; *ER* estrogen receptor; *PR* progesterone; *NACT* neoadjuvant chemotherapy; *AOR* adjusted odds ratio; *CI* confidence interval

a NACT cohort included patients who started NACT for 12–30 weeks before surgery

b *P* values were computed by using Pearson’s chi-squared or Fisher’s exact tests

c Adjusted for age, race/ethnicity, primary payer, facility type, Charlson-Deyo Comorbidity Index, histologic type, HER2 IHC score, clinical T/N stage, and tumor grade

d per 10-unit increase for the AORs

e Adjusted for age, race/ethnicity, primary payer, facility type, Charlson-Deyo Comorbidity Index, histologic type, PR status, HER2 IHC score, clinical T/N stage, and tumor grade

† *P* < 0.01;

§ *P* < 0.001

**TABLE 3:** Associated characteristics with surgical plan (breast-conserving surgery vs. mastectomy) in early-stage, HR-positive/HER2-negative breast cancer patients who received neoadjuvant endocrine therapy

Overall	Among patients who received NET only^a^			
	Surgical plan		*P* ^b^	Multivariable logistic regression
	Mastectomy	Breast-conserving surgery		
	n = 3555 (36.0%)	n = 6314 (64.0%)		
	No. (%)	No. (%)		AOR (95% CI)
Age at diagnosis				
Mean (SD)	68.4 (11.7)	69.2 (11.1)	<0.001	**1.16 (1.09–1.23)** ^§,d^
Median (IQR)	69.0 (60.0, 77.0)	69.0 (62.0, 77.0)	0.009	
Age group (years)				
≤50	294 (8.3)	313 (5.0)	<0.001	–
>50	3261 (91.7)	6001 (95.0)		–
Race/ethnicity^c^				
Non-Hispanic White	2695 (75.8)	4955 (78.5)	0.010	1.0 (reference)
Non-Hispanic Black	410 (11.5)	608 (9.6)		0.89 (0.75–1.05)
Non-Hispanic Asian	135 (3.8)	199 (3.2)		**0.69 (0.53–0.91)** ^#^
Hispanic	256 (7.2)	439 (7.0)		1.01 (0.83–1.24)
Other	59 (1.7)	113 (1.8)		1.18 (0.79–1.76)
Primary payer at diagnosis				
Uninsured	116 (3.3)	127 (2.0)	<0.001	**0.59 (0.43–0.82)** ^†^
Private/managed care	1047 (29.5)	1875 (29.7)		1.0 (reference)
Medicaid	253 (7.1)	342 (5.4)		0.85 (0.68–1.07)
Medicare	2073 (58.3)	3849 (61.0)		0.93 (0.81–1.08)
Other government/unknown	66 (1.9)	121 (1.9)		0.95 (0.64–1.42)
Facility type/cancer program				
Community	179 (5.1)	352 (5.6)	0.680	1.18 (0.93–1.51)
Comprehensive community	1172 (33.3)	2112 (33.6)		1.07 (0.95–1.20)
Academic/research	1409 (40.0)	2476 (39.4)		1.0 (reference)
Integrated network	762 (21.6)	1339 (21.3)		0.99 (0.86–1.14)
Charlson-Deyo Comorbidity Index				
0	2721 (76.5)	4931 (78.1)	0.120	1.0 (reference)
1	550 (15.5)	883 (14.0)		**0.85 (0.73–0.98)**
≥2	284 (8.0)	500 (7.9)		0.90 (0.74–1.08)
Histologic type				
Ductal	2033 (57.2)	4474 (70.9)	<0.001	1.0 (reference)
Lobular	1061 (29.8)	1128 (17.9)		**0.55 (0.49–0.63)** ^§^
Ductal and lobular	271 (7.6)	414 (6.6)		**0.71 (0.58–0.87)** ^†^
Other	190 (5.3)	298 (4.7)		0.80 (0.63–1.03)
AJCC stage group				
I	859 (24.2)	2836 (44.9)	<0.001	–
II	1754 (49.3)	3061 (48.5)		–
III	942 (26.5)	417 (6.6)		–
AJCC clinical T stage				
cT1	630 (17.8)	2154 (34.2)	<0.001	1.0 (reference)
cT2	1433 (40.4)	3369 (53.4)		**0.78 (0.69–0.88)** ^§^
cT3	876 (24.7)	526 (8.3)		**0.24 (0.21–0.29)** ^§^
cT4	610 (17.2)	257 (4.1)		**0.14 (0.12–0.18)** ^§^
AJCC clinical nodal status				
Negative (cN0)	2432 (69.0)	5360 (85.3)	<0.001	**1.96 (1.73–2.22)**
Positive (cN1+)	1093 (31.0)	924 (14.7)		1.0 (reference)
PR status				
Negative	463 (13.1)	597 (9.5)	<0.001	**0.72 (0.61–0.85)** ^§^
Positive	3084 (86.9)	5710 (90.5)		1.0 (reference)
HER2 IHC				
Score 0	900 (30.5)	1598 (30.3)	0.790	1.0 (reference)
Score 1+	1374 (46.6)	2497 (47.3)		0.95 (0.84–1.07)
Score 2+	677 (22.9)	1184 (22.4)		0.90 (0.79–1.04)
Tumor grade				
1	915 (27.6)	1862 (30.8)	<0.001	**1.26 (1.05–1.51)**
2	1942 (58.6)	3545 (58.7)		**1.22 (1.04–1.44)**
3	459 (13.8)	631 (10.5)		1.0 (reference)
Days of NET surgery				
Mean (SD)	216.5 (95.8)	221.2 (101.1)	0.025	–
Median (IQR)	190.0 (154.0, 248)	192.0 (155.0, 251.0)	0.170	–

Bold values indicate significant findings at *P* < 0.05

*HR* hormone receptor; *HER2* human epidermal growth factor receptor 2; *NET* neoadjuvant endocrine therapy; *SD* standard deviation; *IQR* interquartile range; *PR* progesterone receptor; *IHC* immunohistochemistry; *AJCC* American Joint Committee on Cancer; *AOR* adjusted odds ratio; *CI* confidence interval

a NET cohort included patient who started NET within 4 months to 3 years before surgery

b *P* values were computed using Student’s *t*, Wilcoxon rank-sum, or Pearson’s chi-squared tests as appropriate

c Other includes American Indian, Alaska Native, Hawaiian and other Pacific Islander, other or unknown races/ethnicities

d AOR per 10-year increase for age at diagnosis

† *P* < 0.005;

# *P* < 0.01;

§ *P* < 0.001

**TABLE 4:** Subgroup analyses of surgical plan in early-stage, HR-positive/HER2-negative breast cancer patients who received neoadjuvant endocrine therapy, with available quantitative biomarker and genomic assay data

	Among patients who received NET only^a^			
	Surgical plan		*P* ^b^	Multivariable logistic regression
	Mastectomy	Breast-conserving surgery		
	No. (row %)	No. (row %)		AOR (95% CI)
ER%, mean (SD)	92.7 (11.6)	94.0 (10.1)	<0.001	**1.09 (1.01–1.17)** ^c,d^
PR%, mean (SD)	60.1 (37.4)	62.6 (36.8)	0.055	1.02 (0.99–1.04)^c,d^
PR% category				
0%	152 (39.0)	238 (61.0)	0.105	1.0 (reference)
1–10%	97 (31.3)	213 (68.7)		**1.46 (1.01–2.10)** ^c^
>10%	3306 (36.1)	5863 (63.9)		**1.30 (1.02–1.66)** ^c^
Ki-67%, mean (SD)	18.8 (17.0)	18.0 (16.3)	0.306	**0.98 (0.91–1.05)** ^c,d^
Ki-67%, category				
<10%	210 (31.4)	458 (68.6)	0.038	1.19 (0.92–1.54)^c^
10–20%	261 (36.1)	462 (63.9)		1.0 (reference)
>20%	3084 (36.4)	5394 (63.6)		**1.21 (1.01–1.45)** ^c^
21-gene recurrence score				
Low (0–10)	155 (31.4)	338 (68.6)	0.768	0.92 (0.60–1.43)^e^
Intermediate (11–25)	453 (33.1)	914 (66.9)		0.96 (0.65–1.41)^e^
High (26–100)	67 (31.9)	143 (68.1)		1.0 (reference)
70-gene risk group				
Low risk	93 (35.6)	168 (64.4)	0.651	1.0 (reference)
High risk	28 (32.9)	57 (67.1)		0.94 (0.43–2.04)^e^

Bold values indicate significant findings at *P* < 0.05

*HR* hormone receptor; *HER2* human epidermal growth factor receptor 2; *ER* estrogen receptor; *PR* progesterone; *NET* neoadjuvant endocrine therapy; *AOR* adjusted odds ratio; *CI* confidence interval

a NET cohort included patient who started NET within 4 months to 3 years before surgery

b *P* values were computed by using Pearson’s chi-squared or Fisher’s exact tests

c Adjusted for age, race/ethnicity, primary payer, facility type, Charlson-Deyo Comorbidity Index, histologic type, HER2 IHC score, clinical T/N stage, and tumor grade

d Per 10-unit increase for the AORs

e Adjusted for age, race/ethnicity, primary payer, facility type, Charlson-Deyo Comorbidity Index, histologic type, PR status, HER2 IHC score, clinical T/N stage, and tumor grade

† *P* < 0.01;

§ *P* < 0.001

## Data Availability

Data for this study were obtained from the National Cancer Database (NCDB). Investigators affiliated with Commission on Cancer-accredited cancer programs can request the NCDB data file by submitting an application to the American College of Surgeons (ACS) via https://www.facs.org/quality-programs/cancer-programs/national-cancer-database.
