# Supplementary material for: Quantitative Biomarkers, Genomic Assays, and Demographics Associated with Breast-Conserving Surgery Following Neoadjuvant Therapy in Early-Stage, Hormone Receptor-Positive, HER-Negative Breast Cancer
Source: Ann Surg Oncol. Author manuscript; Available in PMC 2025 Dec 1. (PMC11549201; doi:10.1245/s10434-024-16160-5)
Supplement: Supplemental Materias [file NIHMS2022297-supplement-Supplemental_Materias.pdf]

Supplemental Materials

Supplementary Figure 1. Supplementary Figure 1. CONSORT diagram of cohort selection of patients with early-stage, HR-positive/HER2-negative breast cancer who received neoadjuvant systemic therapy

Supplementary Figure 2. Estimated rates of pathologic complete response by days of neoadjuvant systemic therapy prior to surgery using restricted cubic spline logistic regression

Supplementary Table 1. Characteristics of patients with early-stage, HR-positive/HER2-negative breast cancer overall and by neoadjuvant therapy

Supplementary Table 2. Percentages of surgical plans among patients with early-stage, HR-positive/HER2-negative breast cancer

Supplementary Table 3. Characteristics of patients with early-stage, HR-positive/HER2-negative breast cancer by pathological complete response

Supplementary Figure 3. Estimated rates of breast-conserving surgery in early-stage, HR-positive/HER2-negative, cT3-4 breast cancer by quantitative biomarkers and 21-gene recurrence scores

Supplementary Figure 1. CONSORT diagram of cohort selection of patients with early-stage, HR-positive/HER2-negative breast cancer who received neoadjuvant systemic therapy

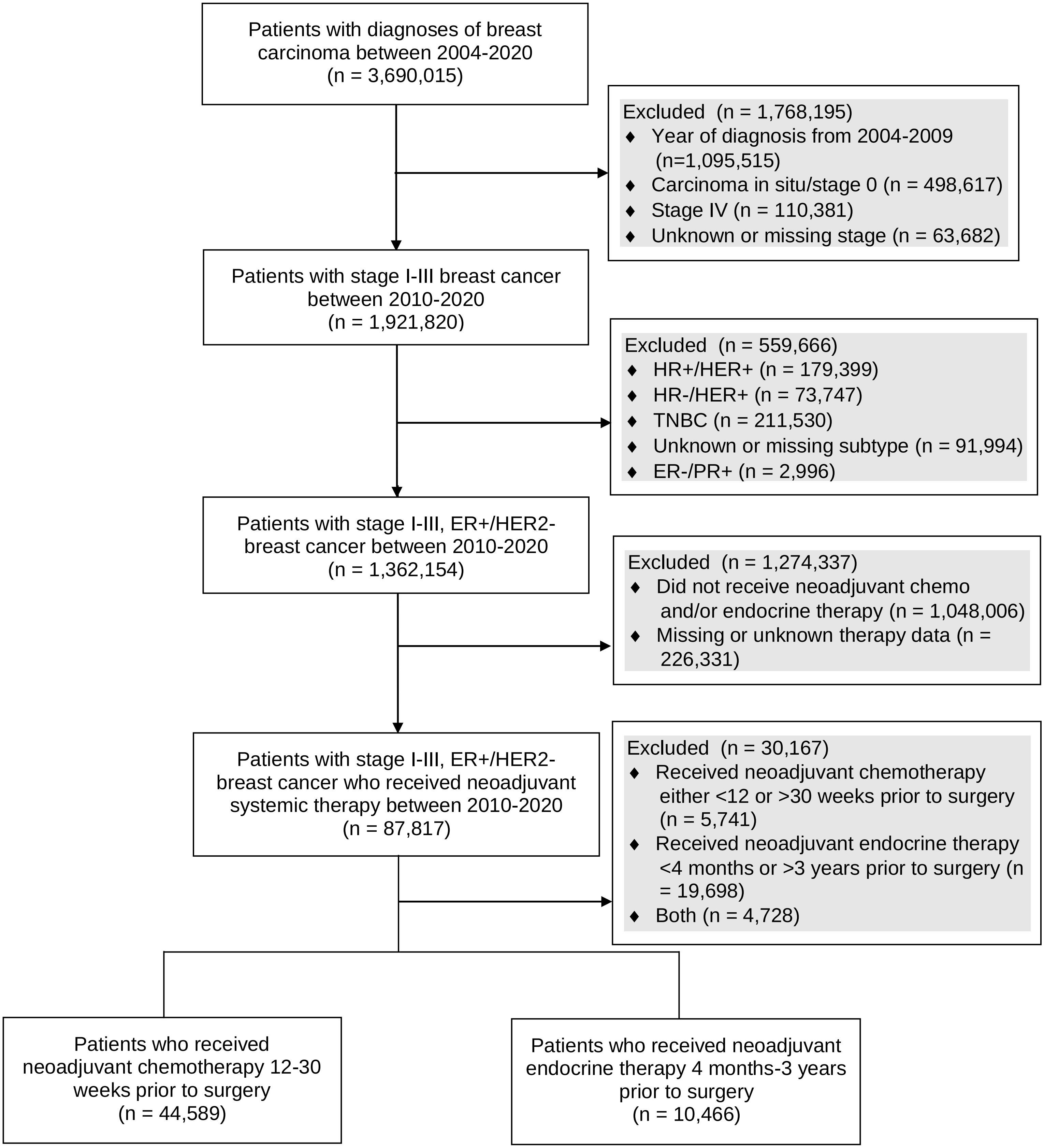

Supplementary Figure 2. Estimated rates of pathologic complete response by days of neoadjuvant systemic therapy prior to surgery using restricted cubic spline logistic regression

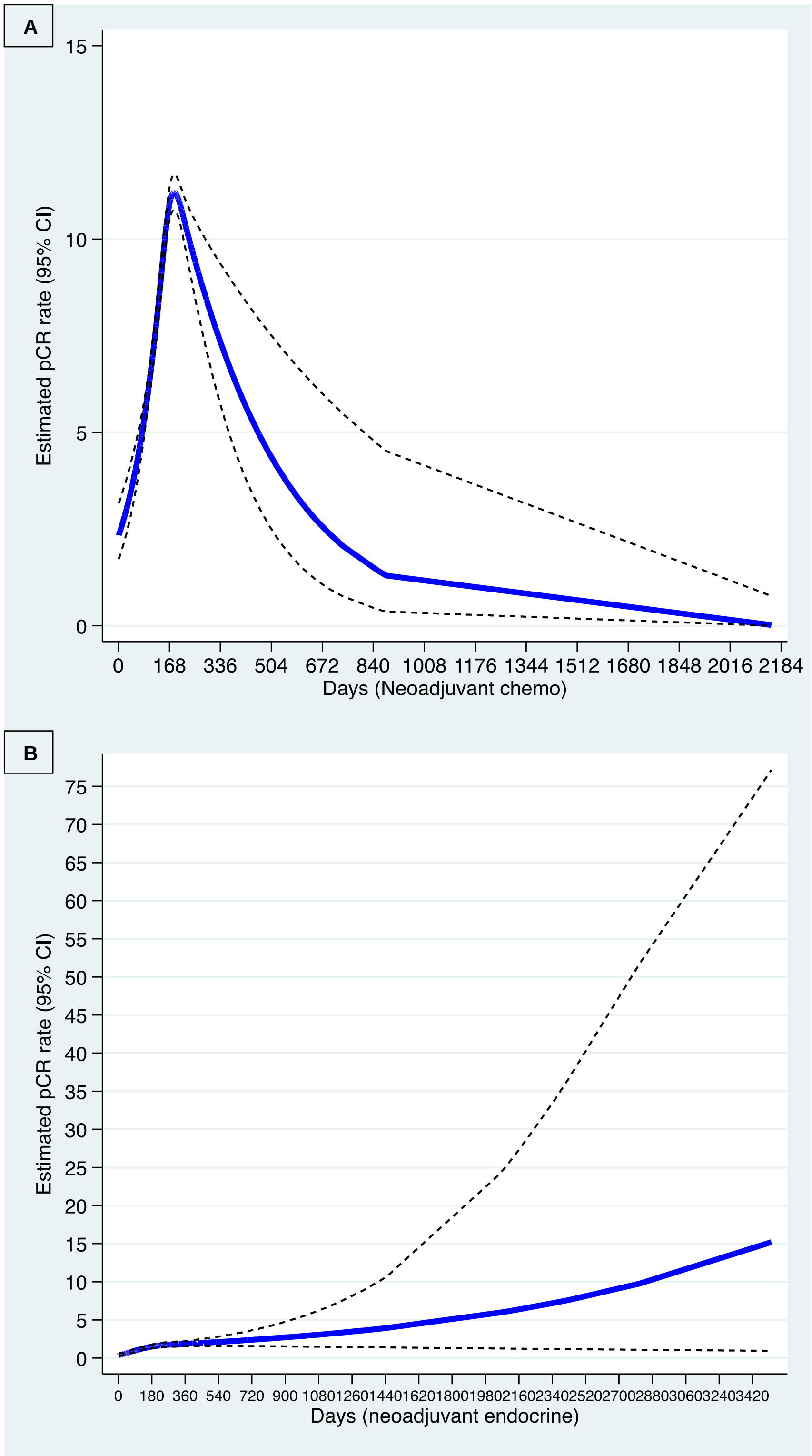

The blue lines represent estimated rates of pathologic complete response, and the dash lines represent the corresponding 95% confidence intervals.

Supplementary Table 1. Characteristics of patients with early-stage, HR-positive/HER2-negative breast cancer overall and by neoadjuvant therapy

**Variable**	**NACT only** [a]	**NET only** [b]	
	n = 44589 (81.0%)	n = 10466 (19.0%)	
	**No. (%)**	**No. (%)**	***P* value** [c]
**Age at diagnosis**			
Mean (SD)	53.1 (11.8)	68.4 (11.5)	<0.001
**Age group**			
≤50 years	19075 (42.8)	728 (7.0)	<0.001
>50 years	25514 (57.2)	9738 (93.0)	
**Sex assigned at birth**			
Male	373 (0.8)	83 (0.8)	0.659
Female	44216 (99.2)	10383 (99.2)	
**Race/ethnicity** [d]			
Non-Hispanic White	30829 (69.1)	8120 (77.6)	<0.001
Non-Hispanic Black	6395 (14.3)	1080 (10.3)	
Non-Hispanic Asian	2011 (4.5)	351 (3.4)	
Hispanic	4283 (9.6)	728 (7.0)	
Other	1071 (2.4)	187 (1.8)	
**Primary payer at diagnosis**			
Uninsured	1643 (3.7)	255 (2.4)	<0.001
Private/managed care	28256 (63.4)	3231 (30.9)	
Medicaid	5198 (11.7)	648 (6.2)	
Medicare	8307 (18.6)	6129 (58.6)	
Other government/unknown	1185 (2.7)	203 (1.9)	
**Facility type/cancer program**			
Community	2452 (6.4)	549 (5.3)	<0.001
Comprehensive community	15079 (39.1)	3475 (33.5)	
Academic/research	12857 (33.4)	4146 (39.9)	
Integrated network	8135 (21.1)	2214 (21.3)	
**Year of diagnosis**			
2010	2861 (6.4)	435 (4.2)	<0.001
2011	3262 (7.3)	557 (5.3)	
2012	3470 (7.8)	574 (5.5)	
2013	3748 (8.4)	696 (6.7)	
2014	4134 (9.3)	781 (7.5)	
2015	4237 (9.5)	1014 (9.7)	
2016	4596 (10.3)	1161 (11.1)	
2017	4529 (10.2)	1245 (11.9)	
2018	4639 (10.4)	1278 (12.2)	
2019	4690 (10.5)	1188 (11.4)	
2020	4423 (9.9)	1537 (14.7)	
**Charlson-Deyo Comorbidity Index**			
0	38811 (87.0)	8145 (77.8)	<0.001

1	4621 (10.4)	1512 (14.4)	
≥2	1157 (2.6)	809 (7.7)	
**Histologic type**			
Ductal	34602 (77.6)	6840 (65.4)	<0.001
Lobular	4663 (10.5)	2382 (22.8)	
Ductal and lobular	2106 (4.7)	730 (7.0)	
Other	3218 (7.2)	514 (4.9)	
**AJCC stage group**			
I	6140 (13.8)	3883 (37.1)	<0.001
II	24131 (54.1)	5108 (48.8)	
III	14318 (32.1)	1475 (14.1)	
**ER%**			
Mean (SD)	74.2 (34.5)	93.5 (10.7)	<0.001
Median (IQR)	94.0 (65.0, 96.0)	95.0 (94.0, 100.0)	<0.001
**PR%**			
Mean (SD)	44.0 (40.0)	62.1 (36.8)	<0.001
Median (IQR)	40.0 (0.0, 90.0)	80.0 (25.0, 95.0)	<0.001
**HER2 IHC**			
Score 0	10523 (29.3)	2645 (30.3)	<0.001
Score 1+	15877 (44.3)	4117 (47.2)	
Score 2+	9476 (26.4)	1963 (22.5)	
**Ki-67%**			
Mean (SD)	39.7 (27.3)	18.1 (16.4)	<0.001
Median (IQR)	33.0 (17.9, 60.0)	14.0 (6.0, 25.0)	<0.001
**AJCC clinical T stage**			
cT1	7926 (17.9)	2916 (27.9)	<0.001
cT2	20940 (47.2)	5073 (48.5)	
cT3	9645 (21.7)	1540 (14.7)	
cT4	5852 (13.2)	923 (8.8)	
**AJCC clinical nodal status**			
Negative (cN0)	18873 (42.6)	8223 (79.0)	<0.001
Positive (cN1+)	25460 (57.4)	2181 (21.0)	
**Tumor grade**			
1	4225 (10.1)	2956 (29.8)	<0.001
2	19741 (47.1)	5822 (58.7)	
3	17975 (42.9)	1145 (11.5)	
**21-gene recurrence score**			
Mean (SD)	31.0 (16.0)	16.1 (8.4)	<0.001
Median (IQR)	29.0 (21.0, 38.0)	15.0 (11.0, 21.0)	<0.001
Low (0-10)	115 (6.6)	533 (23.8)	<0.001
Intermediate (11-25)	503 (28.9)	1489 (66.4)	
High (26-100)	1120 (64.4)	222 (9.9)	
**70-gene risk group**			
Low risk	142 (16.1)	296 (76.1)	<0.001

High risk	741 (83.9)	93 (23.9)	
**Days of cancer treatment prior to surgery**			
Mean (SD)	150.0 (29.7)	218.1 (98.2)	
Median (IQR)	152.0 (130.0, 173.0)	190.0 (154.0, 247.0)	

Abbreviations: HR, hormone receptor; HER2, human epidermal growth factor receptor 2; No., number; NACT, neoadjuvant chemotherapy; NET, neoadjuvant endocrine therapy; SD, standard deviation; IQR, interquartile range; ER, estrogen receptor; PR, progesterone receptor; IHC, immunohistochemistry; AJCC, American Joint Committee on Cancer.

[a] The NACT cohort included patients who started NACT for 12-30 weeks prior to surgery.

[b] The NET cohort included patient who started NET within 4 month to 3 years prior to surgery.

[c] *P* values were computed using Student’s t, Wilcoxon rank-sum, or Chi-squared tests as appropriate.

[d] Other includes American Indian, Alaska Native, Hawaiian and other Pacific Islander, other or unknown races/ethnicities.

Supplementary Table 2. Percentages of surgical plans among patients with early-stage, HR-positive/HER2-negative breast cancer

		Surgical plan		
		**Mastectomy**	**Breast-conserving surgery**	
		n = 22566 (52.7%)	n=20247 (47.3%)	
	**Neoadjuvant therapy**	**No. (row %)**	**No. (row %)**	***P* value [a]**
**Overall**				
	NACT only [b]	19011 (57.7)	13933 (42.3)	<0.001
	NET only [c]	3555 (36.0)	6314 (64.0)	
**Patients who achieved pCR**				
	NACT only [b]	1291 (43.0)	1710 (57.0)	0.013
	NET only [c]	48 (32.7)	99 (67.3)	
**Patients who did not achieve pCR**				
	NACT only [b]	17720 (59.2)	12223 (40.8)	<0.001
	NET only [c]	3507 (36.1)	6215 (63.9)	

Abbreviations: HR, hormone receptor; HER2, human epidermal growth factor receptor 2; No., number; NACT, neoadjuvant chemotherapy; NET, neoadjuvant endocrine therapy; pCR, pathological complete response.

[a] *P* value was computed using the Pearson's chi-squared test.

[b] The NACT cohort included patients who started NACT for 12-30 weeks prior to surgery.

[c] The NET cohort included patient who started NET within 4 month to 3 years prior to surgery.

Supplementary Table 3. Characteristics of patients with early-stage, HR-positive/HER2-negative breast cancer by pathological complete response

	Pathological complete response		
	**Did not achieve**	**Achieved**	
	**No. (%)**	**No. (%)**	***P* value** [a]
**Age at diagnosis**			
Mean (SD)	56.4 (13.3)	50.9 (12.3)	<0.001
Median (IQR)	56.0 (47.0, 66.0)	50.0 (42.0, 60.0)	<0.001
**Age group**			
≤50 years	18524 (34.8)	2268 (50.5)	<0.001
>50 years	34639 (65.2)	2219 (49.5)	
**Sex assigned at birth**			
Male	456 (0.9)	16 (0.4)	<0.001
Female	52707 (99.1)	4471 (99.6)	
**Race/Ethnicity** [b]			
Non-Hispanic White	37809 (71.1)	2925 (65.2)	<0.001
Non-Hispanic Black	7061 (13.3)	763 (17.0)	
Non-Hispanic Asian	2291 (4.3)	221 (4.9)	
Hispanic	4779 (9.0)	488 (10.9)	
Other	1223 (2.3)	90 (2.0)	
**Primary payer at diagnosis**			
Uninsured	1822 (3.4)	155 (3.5)	<0.001
Private/managed care	29941 (56.3)	3011 (67.1)	
Medicaid	5656 (10.6)	493 (11.0)	
Medicare	14402 (27.1)	725 (16.2)	
Other government/unknown	1342 (2.5)	103 (2.3)	
**Facility type/cancer program**			
Community	2956 (6.2)	189 (5.2)	0.086
Comprehensive community	17865 (37.7)	1384 (38.2)	
Academic/research	16553 (35.0)	1267 (35.0)	
Integrated network	9953 (21.0)	785 (21.7)	
**Year of diagnosis**			
2010	3230 (6.1)	165 (3.7)	<0.001
2011	3714 (7.0)	222 (4.9)	
2012	3892 (7.3)	260 (5.8)	
2013	4200 (7.9)	358 (8.0)	
2014	4721 (8.9)	380 (8.5)	
2015	5025 (9.5)	461 (10.3)	
2016	5570 (10.5)	491 (10.9)	
2017	5586 (10.5)	515 (11.5)	
2018	5704 (10.7)	538 (12.0)	
2019	5687 (10.7)	542 (12.1)	
2020	5834 (11.0)	555 (12.4)	
**Charlson-Deyo Comorbidity**			

**Index**			
0	45242 (85.1)	3936 (87.7)	<0.001
1	5973 (11.2)	432 (9.6)	
≥2	1948 (3.7)	119 (2.7)	
**Histologic type**			
Ductal	39449 (74.2)	3976 (88.6)	<0.001
Lobular	7245 (13.6)	150 (3.3)	
Ductal and lobular	2875 (5.4)	79 (1.8)	
Other	3594 (6.8)	282 (6.3)	
**AJCC stage group**			
I	9548 (18.0)	924 (20.6)	<0.001
II	28075 (52.8)	2552 (56.9)	
III	15540 (29.2)	1011 (22.5)	
**ER %**			
Mean (SD)	82.3 (28.2)	42.9 (40.3)	<0.001
Median (IQR)	95.0 (85.0, 98.0)	23.0 (5.0, 90.0)	<0.001
**PR status**			
Negative	9564 (18.0)	2232 (49.8)	<0.001
Positive	43526 (82.0)	2249 (50.2)	
**PR %**			
Mean (SD)	51.0 (39.5)	19.4 (33.1)	<0.001
Median (IQR)	60.0 (5.0, 90.0)	0.0 (0.0, 25.0)	<0.001
**HER2 IHC**			
Score 0	12576 (29.1)	1236 (34.6)	<0.001
Score 1+	19445 (45.0)	1508 (42.3)	
Score 2+	11201 (25.9)	824 (23.1)	
**Ki-67 %**			
Mean (SD)	32.5 (25.3)	59.1 (29.0)	<0.001
Median (IQR)	25.0 (11.1, 50.0)	65.0 (37.0, 85.0)	<0.001
**AJCC clinical T stage**			
cT1	10103 (19.1)	1156 (25.9)	<0.001
cT2	24863 (47.0)	2376 (53.2)	
cT3	11126 (21.0)	628 (14.1)	
cT4	6835 (12.9)	307 (6.9)	
**AJCC clinical nodal status**			
Negative (cN0)	25872 (49.0)	2581 (57.6)	<0.001
Positive (cN1+)	26967 (51.0)	1898 (42.4)	
**Tumor grade**			
1	7251 (14.5)	254 (6.0)	<0.001
2	25950 (51.8)	900 (21.3)	
3	16898 (33.7)	3069 (72.7)	
**21-gene recurrence score**			
Mean (SD)	22.3 (13.6)	39.6 (18.8)	<0.001
Median (IQR)	20.0 (13.0, 29.0)	37.0 (27.0, 53.0)	<0.001

Low (0-10)	659 (15.9)	14 (6.7)	<0.001
Intermediate (11-25)	2109 (50.8)	30 (14.4)	
High (26-100)	1384 (33.3)	165 (78.9)	
**70-gene risk group**			
Low risk	452 (34.4)	<10 (<9.0)	<0.001
High risk	863 (65.6)	<70 (<92.0)	
**Days of NACT prior to surgery**			
Mean (SD)	149.4 (29.8)	155.5 (27.5)	<0.001
Median (IQR)	151.0 (129.0, 172.0)	158.0 (135.0, 176.0)	<0.001
**Days of NET prior to surgery**			
Mean (SD)	217.6 (97.5)	248.5 (133.1)	<0.001
Median (IQR)	190.0 (154.0, 247.0)	210.0 (165.0, 282.0)	<0.001
**Surgical plan**			
Mastectomy	22326 (53.7)	1380 (42.5)	<0.001
Breast-conserving surgery	19272 (46.3)	1866 (57.5)	

Abbreviations: HR, hormone receptor; HER2, human epidermal growth factor receptor 2; No., number; NACT, neoadjuvant chemotherapy; NET, neoadjuvant endocrine therapy; SD, standard deviation; IQR, interquartile range; ER, estrogen receptor; PR, progesterone receptor; IHC, immunohistochemistry; AJCC, American Joint Committee on Cancer.

[a] *P* values were computed using Student's t, Wilcoxon rank-sum, or Chi-square tests as appropriate.

[b] Other includes American Indian, Alaska Native, Hawaiian and other Pacific Islander, other or unknown races/ethnicities.

Supplementary Figure 3. Estimated rates of breast-conserving surgery in early-stage, HR-positive/HER2-negative, cT3-4 breast cancer by quantitative biomarkers and 21-gene recurrence scores

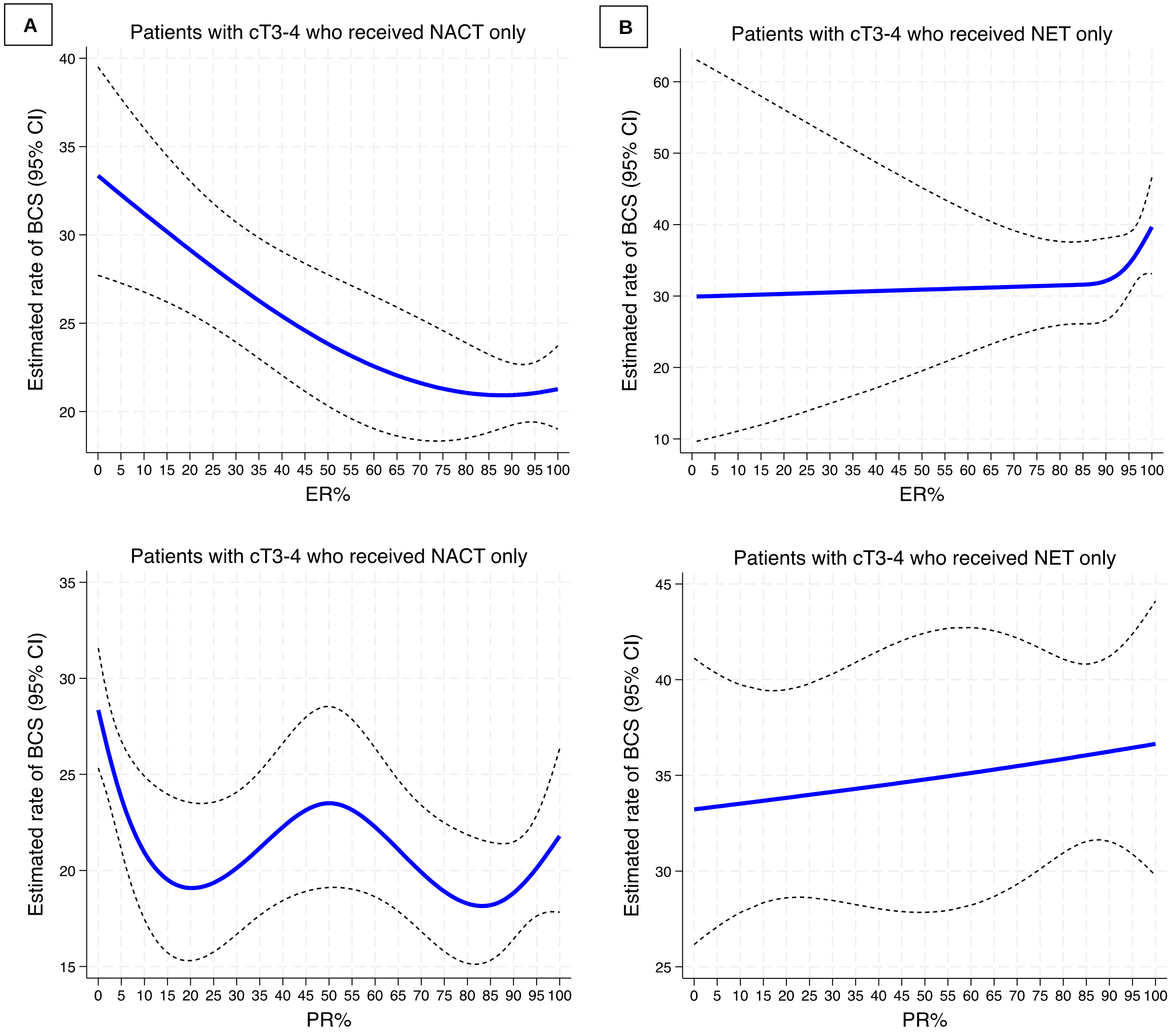

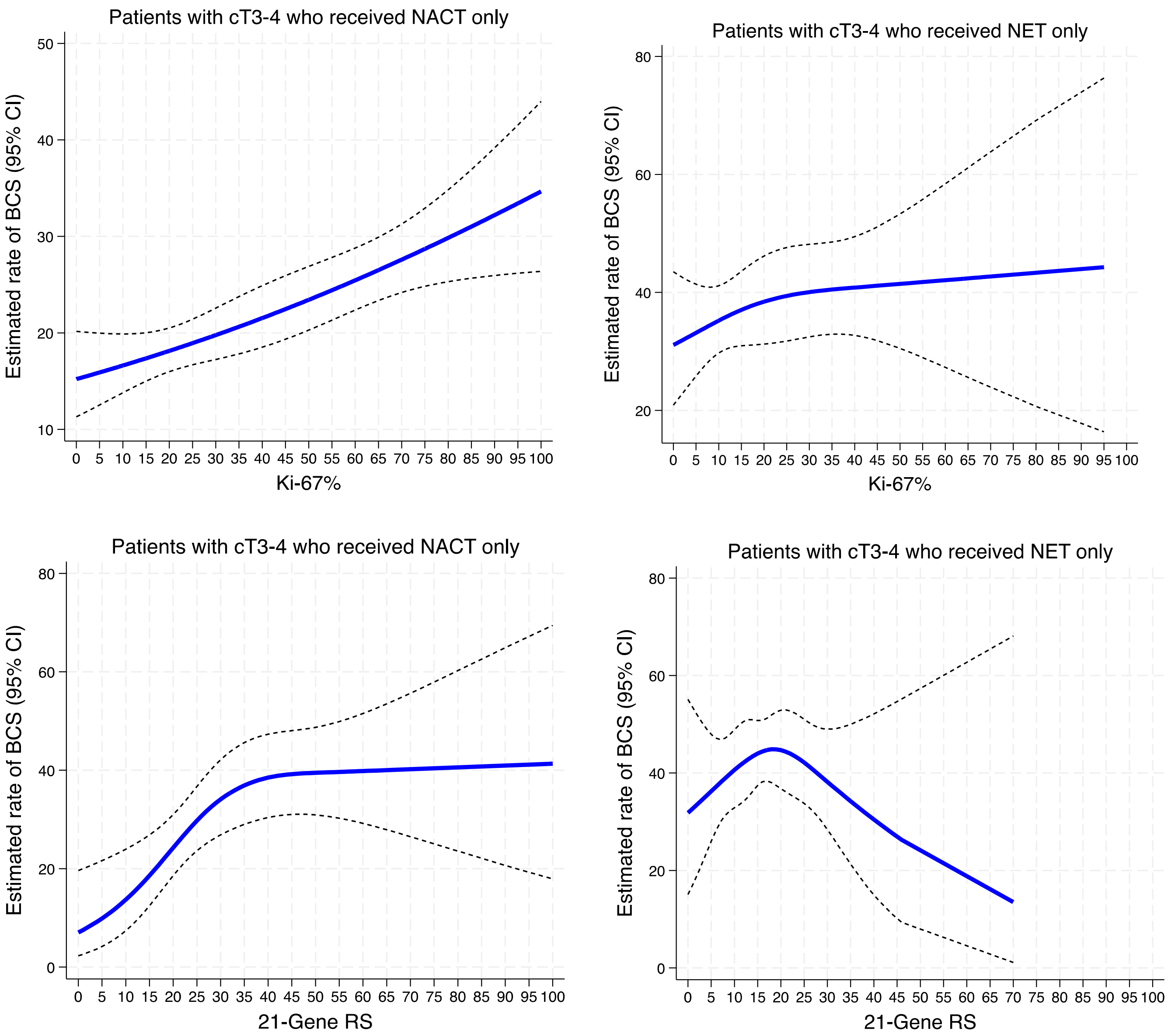

Patients with cT3-4 who received NACT only
Estimated rate of BCS (95% CI)
10
20
30
40
50
0 5 10 15 20 25 30 35 40 45 50 55 60 65 70 75 80 85 90 95 100
Ki-67%
Patients with cT3-4 who received NET only
Estimated rate of BCS (95% CI)
20
40
60
80
0 5 10 15 20 25 30 35 40 45 50 55 60 65 70 75 80 85 90 95 100
Ki-67%
Patients with cT3-4 who received NACT only
Estimated rate of BCS (95% CI)
0
20
40
60
80
0 5 10 15 20 25 30 35 40 45 50 55 60 65 70 75 80 85 90 95 100
21-Gene RS
Patients with cT3-4 who received NET only
Estimated rate of BCS (95% CI)
0
20
40
60
80
0 5 10 15 20 25 30 35 40 45 50 55 60 65 70 75 80 85 90 95 100
21-Gene RS

The blue lines represent estimated rates of breast-conserving surgery, and the dash lines represent the corresponding 95% CIs.

Abbreviations: HR, hormone receptor; HER2, human epidermal growth factor receptor 2; NACT, neoadjuvant chemotherapy; NET, neoadjuvant endocrine therapy; CI, confidence interval; HR, estrogen receptor; PR, progesterone receptor; RS, recurrence score.
